# Adrenocortical carcinoma complicated by renal thrombotic microangiopathy, a case-series

**DOI:** 10.1186/s12882-020-1703-5

**Published:** 2020-01-30

**Authors:** Tristan de Nattes, Lucile Moreau-Grangé, Delphine Vezzosi, Julien Haddoux, Miguel Hie, Dominique Guerrot, Steven Grangé

**Affiliations:** 1 Nephrology – Kidney Transplant Unit, Rouen University Hospital, 76031 Rouen, France; 2 Endocrine Unit, Rouen University Hospital, Rouen, France; 3 Endocrine Unit, Toulouse University Hospital, Toulouse, France; 4 Medical Oncology Department, Gustave Roussy Institute, Villejuif, France; 5 Department of Internal Medicine, Pitie-Salpetriere Hospital, AP-HP, Paris, France; 6 Department of Medical Critical Care, Rouen University Hospital, Rouen, France

**Keywords:** Thrombotic microangiopathy, Hemolytic uremic syndrome, Acute kidney injury, Adrenocortical carcinoma

## Abstract

**Background:**

Cancer-related thrombotic microangiopathy (CR-TMA) is a rare entity associated with a dismal prognosis. Usually, CR-TMA is associated with mucin-producing carcinomas among which stomach, breast, prostate, lung and pancreas tumours are the most frequent.

**Cases presentation:**

We describe for the first time three cases of CR-TMA due to adrenocortical carcinoma (ACC). All of them had mechanical hemolytic anemia and thrombocytopenia without any other identifiable cause. Bicytopenia was diagnosed either simultaneously with ACC or at the time of metastatic evolution. Two patients had acute kidney injury (AKI) with severe pathological findings on kidney biopsy. Despite total adrenalectomy, chemotherapy, and specific treatment of TMA with plasma-exchanges, renal failure and hemolytic anemia remained. The only manifestation of CR-TMA in the third patient was hemolytic anemia, which resolved after surgical removal of ACC. The evolutions in these patients suggests ACC-related TMA may be related to a circulating factor.

**Conclusions:**

CR-TMAs are rare. Here we describe the first case series of ACC-related TMA, among which two had renal involvement. This entity is associated with dismal renal prognosis despite specific treatment of TMA. According to patients’ evolution, the persistence of TMA may reflect an uncontrolled malignancy.

## Background

Thrombotic microangiopathy (TMA) is a rare entity due to a wide variety of diseases, all characterized by mechanical hemolytic anemia, thrombocytopenia and microvascular occlusions. Causes of TMA are separated between thrombotic thrombocytopenic purpura, typical Hemolytic uremic syndrome (HUS), primary atypical HUS in patients with an abnormality of the alternative complement pathway, and HUS secondary to a heterogeneous group of causes: infections, drugs, genetic disorders, systemic diseases and cancers [1]. Adrenocortical carcinoma (ACC) is a rare tumour, with an unfavourable prognosis. Hormonal complications often occur, but paraneoplastic syndromes are uncommon in the literature [2]. Here, we describe a case-series of ACC-associated TMA.

## Cases presentation

### Case 1

A 41-year-old woman was investigated in July 2016 for fatigue and loss of 10 kg over a few weeks. She was not taking any medication at this time and had no history of kidney disease. An abdominal computed tomography scan was performed in July, revealing a 13x13x10cm left adrenal mass of heterogeneous density with heterogeneous and prolonged enhancement after contrast agent administration. This mass was suggestive of ACC not associated with significant hormonal hypersecretion. Other lab results revealed plasma creatinine = 97 μmol/L, proteinuria = 0.7 g/g of urine creatinine, haemoglobin 9.9 g/dL, platelet 143 G/L.

Surgical procedure was planned in early-August, and no treatment was introduced.

At admission, she had stage 3 KDIGO AKI with plasma creatinine at 439 μmol/L and 0.99 g/24 h proteinuria. Urine sediment was normal. Blood pressure was 119/70 mmHg, there was no clinical sign of other organ involvement. She presented thrombocytopenia (67G/L) and mechanical hemolytic anemia: haemoglobin = 7.9 g/dL, reticulocyte = 175G/L, presence of schistocytes, Lactate Dehydrogenase (LDH) = 1058 UI/L and haptoglobin < 0.1 g/L. Extensive laboratory investigations were performed for the differential diagnosis of cytopenias. Antinuclear factors were negative, as were antibodies for antiphospholipid syndrome and scleroderma. ADAMTS 13 activity was 93%, complement investigations were normal (C4, C4, CH50, Factor I and H, anti-Factor H antibody).

The specific treatment of TMA consisted in daily plasma-exchange therapy of 60 mL/kg with plasma as replacement fluid. Intermittent hemodialysis was started on the same day. Left adrenalectomy was performed with splenectomy because of peroperative splenic decapsulation. A kidney biopsy was also performed.

**Fig. 1:**
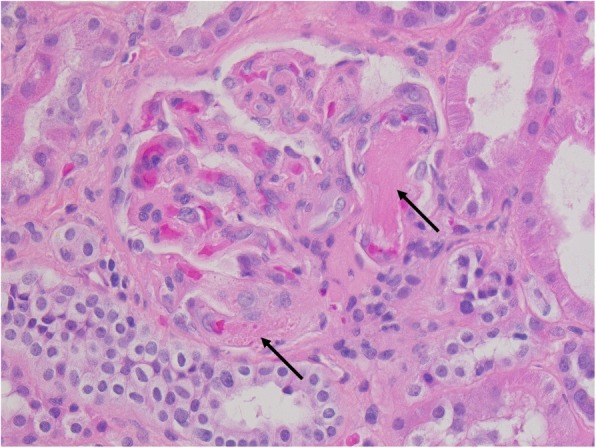
Histologic examination (H&E, × 40). Arrows: fibrin thrombi within glomerular capillary loops

ACC was confirmed by pathology, with a Weiss score at 6/9, Ki67 = 45% and 25 mitotic figures per 50 high-power field. The resection was total, and mesenteric lymphadenopathy was not metastatic, leading to stage III according to ENSAT classification. Renal pathology confirmed TMA. Twenty-six glomeruli were observed, of which 11 were ischemic with fibrin thrombi within glomerular capillary loops. Thrombi were present in interlobular arteries, arterioles and glomerular capillaries with fibrinoid deposit. No duplication of the glomerular basement membranes was found. There was moderate acute tubular necrosis with mild interstitial fibrosis and tubular atrophy (Fig. 1).

Fourteen plasma-exchanges lead to correction of hemolysis, but the patient remained dialysis-dependent, and biological TMA reappeared a few days after plasma-exchange discontinuation. Owing to the isolated renal TMA, unfavourable renal prognosis according to pathology, kidney function evolution, and the lack of efficiency of terminal complement blockade in cancer-related TMA (CR-TMA), we decided not to escalate treatments and to undergo chronic hemodialysis.

Mitotane was started despite lack of pharmacokinetic knowledge on this treatment in hemodialysis patients, with a target plasma concentration between 14 and 20 mg/L. Eighteen months later, metastatic lesions occurred, leading to treatment by etoposide, doxorubicin and cisplatin. During all the follow-up, the patient remained anuric in hemodialysis and had persistent hemolysis with schistocytes, high LDH’s value and decreased haptoglobin. Twenty-four months after diagnosis, she died in a context of severe pneumonia.

### Case 2

A 23-year-old woman was diagnosed with a right ACC in 2014. She first had nephrectomy and adrenalectomy. Despite Mitotane, cerebral, hepatic, pulmonary and bone metastasis occurred, leading to several chemotherapies: Cisplatin, Lenvatinib and Gemcitabine. In October 2018, she presented cardiac tamponade related to influenza-B virus. All investigations at this time did not find any argument for a paraneoplastic aetiology of the pericardial effusion. There was no evidence of TMA and renal function was normal with plasma creatinine 99 μmol/L. Two weeks later, biological data found a KDIGO 3 AKI with plasma creatinine = 200 μmol/L. Hemoglobin was 8.0 g/dL and platelet count 25 G/L. Two weeks later, she had a new cardiac tamponade which required a pericardium-to-pleural space window. Cytopenias were also investigated at this time and revealed TMA with haptoglobin < 0.1 g/L, schistocytes, LDH = 703 UI/L and plasma creatinine = 429 μmol/L. Investigations for this TMA with AKI did not find any other cause of TMA, and metastasis were stable. Due to cytopenias and high suspicious of renal TMA, it was decided not to perform a kidney biopsy.

Daily plasma-exchanges were started for a suspicion of gemcitabine-related TMA. There were no hemodialysis criteria. After 14 plasma-exchanges, platelet count improved (145 G/L), and LDH decreased (422 UI/L) but schistocytes and undetectable haptoglobin persisted. There was no amelioration of kidney function (plasma creatinine 357 μmol/L). After discontinuation of plasma-exchanges, thrombocytopenia recurred. Due to the lack of efficiency of plasma-exchanges and the suspicion of gemcitabine-related TMA, eculizumab was started in December 2018. After second administration of eculizumab, systematic thoracic CT scan revealed new pulmonary metastasis. ACC related-TMA was diagnosed because (1) TMA and kidney function were not ameliorated by discontinuation of gemcitabine and treatment with plasma-exchanges and eculizumab, and (2) recurrent pericarditis with development of new metastatic lesions. Owing to the lack of efficiency of eculizumab in this particular issue, treatment was discontinued and chemotherapy was restarted.

### Case 3

An 81-year-old woman with a medical history of polymyalgia rheumatica and ischemic cardiomyopathy had been hospitalized for the investigation of a bicytopenia which had appeared and worsened over a month. Laboratory results revealed hemolytic anemia (Hb = 9.8 g/dL, haptoglobin < 0.1 g/L and schistocytes) and thrombocytopenia (platelet count 99 G/L). The patient had no organ involvement. Bone marrow morphology confirmed the peripheral origin of the bicytopenia and Coomb’s test was negative, leading to the diagnosis of TMA. Investigations were performed, and an abdominal CT scan revealed a 14 cm partially calcified left adrenal mass, which was not associated with significant hormonal hypersecretion. Left adrenalectomy was performed, with concomitant nephrectomy and splenectomy. Histopathology confirmed ACC with Weiss score at 5/9 and Ki 67 = 40%, ENSAT stage II. The next day, bicytopenia improved and haemolysis resolved, confirming CR-TMA. There was no recurrence of cytopenia during the 2 years follow-up.

## Discussion and conclusion

ACC is a rare tumour, with an incidence of less than 2 cases per million per year and unfavourable prognosis with a 5-year survival rate of 13% in patients with stage-3 ACC [3]. When ACC is suspected, hormonal hypersecretion has to be investigated, especially cortisol, aldosterone and sex steroids. Complete surgical resection has to be performed for all tumours without metastasis [4]. An adjuvant treatment with mitotane is recommended. In patients with advanced disease, systemic chemotherapy with etoposide, doxorubicin and cisplatin is added [5].

In an extensive review, most CR-TMAs were attributed to metastatic adenocarcinomas of gastric, breast, prostate, and lung origin (decreasing frequency). Pathophysiological explanations of CR-TMA remain unclear. One hypothesis is that tumour cell emboli lead to microvascular obstruction, coagulation activation, and vessel wall proliferation. The direct invasion of bone marrow vasculature which results in release of Von Willebrand Factor multimer has also been described [6]. In other cases, activation of the coagulation cascade by mucin released from adenocarcinomas and immune mechanisms has been suggested.

TMA secondary to endocrine malignancies are extremely rare and mainly reported with pheochromocytoma [7]. In those cases, severe hypertension was the most probable explanation of TMA reported by the authors [8–10].

In the first case of ACC-related TMA reported in the present case-series, TMA occurred before any medication, and extensive investigations were negative. In the second case, CR-TMA was treated as gemcitabine-associated TMA which did not improve either cytopenias or renal function. The paraneoplastic aetiology was affirmed by chronology and evolution of illness. In the third case, CR-TMA rapidly improved after total removal of tumor.

The latter case suggests that TMA in this particular situation could be explained by the secretion of a circulating factor by the ACC. This is substantiated by cases 1 and 2, in which plasma-exchange therapy lead to a transient improvement of hemolytic anemia, resuming at plasma-exchange discontinuation. The initial response to plasma-exchange therapy supports the role of a high molecular weight pathogenic factor with endothelial toxicity in the pathophysiology of ACC-related TMA. Considering the improvement of hemolytic anemia with plasma-exchange therapy, the recurrence of cytopenias at plasma-exchange discontinuation despite surgery and chemotherapy may suggest insufficient treatment of the tumor.

Therefore, we suggest that patients with TMA in a context of ACC be treated by plasma-exchange therapy until specific treatment of tumor is performed, with total surgery if possible. Due to the lack of evidence for complement involvement in ACC-related TMA, and since eculizumab efficiency in CR-TMA is not established, we believe complement blockade should not be recommended as a suitable option in this setting.

According to the first two cases, and as it is known for CR-TMA, renal prognosis in ACC-related TMA seems to be dismal. Indeed, despite plasma-exchange therapy, there was no improvement in renal function at the end of the follow-up.

CR-TMAs are rare and associated with dismal prognosis. Here we describe the first case series of ACC-related TMA in 3 different patients. The evolutions in these patients suggests ACC-related TMA may be related to a circulating factor. We therefore suggest plasma-exchange therapy in this context until specific treatment of ACC is initiated.

## Data Availability

The datasets used and/or analysed during the current study available from the corresponding author on reasonable request.

## Abbreviations

AAC: Adrenocortical carcinoma
AKI: Acute Kidney Injury
CR-TMA: Cancer-related Thrombotic Microangiopathy
ENSAT: European Network for the Study of Adrenal Tumours
HUS: Hemolytic uremic syndrome
LDH: Lactate Dehydrogenase
