# Correction to: Adrenocortical carcinoma complicated by renal thrombotic microangiopathy, a case-series

**DOI:** 10.1186/s12882-020-1712-4

**Published:** 2020-02-10

**Authors:** Tristan de Nattes, Lucile Moreau-Grangé, Delphine Vezzosi, Julien Hadoux, Miguel Hie, Dominique Guerrot, Steven Grangé

**Affiliations:** 1 Nephrology – Kidney Transplant Unit, Rouen University Hospital, 76031 Rouen, France; 2 Endocrine Unit, Rouen University Hospital, Rouen, France; 3 Endocrine Unit, Toulouse University Hospital, Toulouse, France; 4 Medical Oncology Department, Gustave Roussy Institute, Villejuif, France; 5 Department of Internal Medicine, Pitie-Salpetriere Hospital, AP-HP, Paris, France; 6 Department of Medical Critical Care, Rouen University Hospital, Rouen, France

**Correction to: BMC Nephrol**

**https://doi.org/10.1186/s12882-020-1703-5**

Following publication of the original article [1], we have been notified that the name of one author was spelled incorrectly as Julien Haddoux, when the correct spelling is Julien Hadoux.

The original article has been corrected.

## References

[1] de Nattes (2020). Adrenocortical carcinoma complicated by renal thrombotic microangiopathy, a case-series. BMC Nephrol.

